# Cancer prognosis and treatment results in patients with PTEN Hamartoma Tumour Syndrome (PHTS)—a European cohort study

**DOI:** 10.1038/s44276-025-00157-y

**Published:** 2025-06-04

**Authors:** Linda A. J. Hendricks, Katja C. J. Verbeek, Janneke H. M. Schuurs-Hoeijmakers, Robin de Putter, Hilde Brems, Sien H. Van Daele, Violetta C. Anastasiadou, Lenka Foretová, Patrick R. Benusiglio, Anna Gerasimenko, Chrystelle Colas, Marie-Charlotte Villy, Claude Houdayer, Maud Branchaud, Robert Hüneburg, Stefan Aretz, Arne Jahn, Verena Steinke-Lange, Giovanni Innella, Daniela Turchetti, Valeria Barili, Maurizio Genuardi, Arianna Panfili, Margherita Baldassarri, Arvīds Irmejs, Mirjam M. de Jong, Thera P. Links, Edward M. Leter, Daniëlle G. M. Bosch, Stephany H. Donze, Rachel S. van der Post, Arjen R. Mensenkamp, Harm Westdorp, Hildegunn Høberg-Vetti, Marianne Tveit Haavind, Kjersti Jørgensen, Lovise Mæhle, Siri Briskemyr, Juliette Dupont Garcia, Ana Blatnik, Judith Balmaña, Maite Torres, Joan Brunet, Roser Lleuger-Pujol, Emma Tham, Marc Tischkowitz, D. Gareth Evans, Zerin Hyder, Nicoline Hoogerbrugge, Janet R. Vos

**Affiliations:** 1 Department of Human Genetics, Radboudumc expert centre for PHTS, Radboud university medical centre, Nijmegen, the Netherlands; 2 Radboud Institute for Medical Innovation, Radboud university medical centre, Nijmegen, the Netherlands; 3 Centre for Medical Genetics, Ghent University Hospital, Ghent, Belgium; 4 European Reference Network for Genetic Tumour Risk Syndromes (ERN GENTURIS), Nijmegen, the Netherlands; 5 Department of Human Genetics, University of Leuven, Leuven, Belgium; 6 Karaiskakio Foundation, Nicosia Cyprus, and Archbishop Makarios III Children’s Hospital, Nicosia, Cyprus; 7 Department of Cancer Epidemiology and Genetics, Masaryk Memorial Cancer Institute, Brno, Czech Republic; 8 Department of Medical Genetics, Pitié-Salpêtrière Hospital, APHP, Sorbonne University, Paris, France; 9 Sorbonne University, Paris Brain Institute - ICM, Inserm, CNRS, Hôpital de la Pitié Salpêtrière, 75013 Paris, France; 10 APHP Sorbonne Université, GH Pitié Salpêtrière et Trousseau, Departement of Genetics, Centre de référence “déficiences intellectuelles de causes rares”, Paris, France; 11 Institut Curie, Service de Génétique, Paris, France; 12 Inserm U830, DNA Repair and Uveal Melanoma (D.R.U.M.), Equipe Labellisée Par la Ligue Nationale Contre le Cancer, Paris, France; 13 Univ Rouen Normandie, Inserm U1245, FHU-G4 Génomique and CHU Rouen, Department of Genetics, 76000 Rouen, France; 14 Centre for Hereditary Tumour Syndromes, University Hospital of Bonn, Bonn, Germany; 15 Department of Internal Medicine I, University Hospital Bonn, Bonn, Germany; 16 Institute of Human Genetics, Medical Faculty, University of Bonn, Bonn, Germany; 17 Institute for Clinical Genetics, Faculty of Medicine Carl Gustav Carus, Technische Universität Dresden, Dresden, Germany; 18 ERN-GENTURIS, Hereditary Cancer Syndrome Centre Dresden, Dresden, Germany; 19 German Cancer Consortium (DKTK), Dresden, Germany; 20 National Centre for Tumour Diseases (NCT), Partner Site Dresden, Dresden, Germany; 21 Medical Genetics Centre, Germany; Arbeitsgruppe Erbliche Gastrointestinale Tumore, Medizinische Klinik und Poliklinik IV - Campus Innenstadt, Klinikum der Universität München, München, Germany; 22 Department of Medical and Surgical Sciences, Centre for Studies on Hereditary Cancer, University of Bologna, Bologna, Italy; 23 Medical Genetics Unit, IRCCS Azienda Ospedaliero-Universitaria di Bologna, Bologna, Italy; 24 Medical Genetics, Department of Medicine and Surgery, University of Parma, Parma, Italy; 25 Department of Laboratory and Infectious Diseases, Fondazione Policlinico Universitario A. Gemelli IRCCS, Rome, Italy; 26 Medical Genetics Section, Department of Life Sciences and Public Health, Università Cattolica del Sacro Cuore, Rome, Italy; 27 Medical Genetics, University of Siena, Siena, Italy; 28 Med Biotech Hub and Competence Centre, Department of Medical Biotechnologies, University of Siena, Siena, 53100 Italy; 29 Genetica Medica, Azienda Ospedaliero-Universitaria Senese, Siena, 53100 Italy; 30 Institute of Oncology, Riga Stradins University, Riga, Latvia; 31 Breast Unit, Pauls Stradins Clinical University Hospital, Riga, Latvia; 32 Department of Genetics, University of Groningen, University Medical Centre Groningen, Groningen, the Netherlands; 33 Department of Endocrinology, University of Groningen, University Medical Centre Groningen, Groningen, the Netherlands; 34 Department of Clinical Genetics, Maastricht University Medical Centre, Maastricht, the Netherlands; 35 Department of Clinical Genetics, Erasmus MC Rotterdam, Rotterdam, the Netherlands; 36 Department of Pathology, Radboud university medical centre, Nijmegen, the Netherlands; 37 Department of Medical Oncology, Radboud university medical centre, Nijmegen, the Netherlands; 38 Western Norway Familial Cancer Centre, Department of Medical Genetics, Haukeland University Hospital, Bergen, Norway; 39 Faculty of Health Studies, VID Specialized University, Bergen, Norway; 40 Department of Medical Genetics, Oslo University Hospital, Oslo, Norway; 41 Department of Medical Genetics, University Hospital of North Norway, Tromsø, Norway; 42 Centro Hospitalar Universitário Lisboa Norte, Lisbon, Portugal; 43 Department of Clinical Cancer Genetics, Institute of Oncology Ljubljana, Ljubljana, Slovenia; 44 Medical Oncology Department, Vall d’Hebron Hospital Universitari, Vall d’Hebron Barcelona Hospital Campus, Barcelona, Spain; 45 Hereditary Cancer Program, Catalan Institute of Oncology, IDIBELL-IDIBGI, Barcelona-, Girona, Spain; 46 Department of Clinical Genetics, Karolinska University Hospital, Stockholm, Sweden; 47 Department of Molecular Medicine and Surgery, Karolinska Institutet, Stockholm, Sweden; 48 Department of Medical Genetics, National Institute for Health Research Cambridge Biomedical Research Centre, University of Cambridge, Cambridge, UK; 49 Manchester Centre for Genomic Medicine, St Mary’s Hospital, Division of Evolution and Genomic Sciences, School of Biological Sciences, University of Manchester, Manchester, UK

## Abstract

**Background:**

PTEN hamartoma tumour syndrome (PHTS) patients have a high hereditary risk of cancer, especially breast (BC), endometrial (EC), and thyroid cancer (TC). However, the prognosis of PHTS-related cancers is unknown.

**Methods:**

This European cohort study included adult PHTS patients with data from medical files, registries, and/or questionnaires. Overall survival (OS) was assessed using Kaplan-Meier analyses and were compared with sporadic cancer and the general population using standardized mortality (SMR) and relative survival rates (RSR). Survival bias was addressed using left-truncation.

**Results:**

Overall, 147 BC patients were included. The 10y-OS was 77% (95%CI = 66–90), decreasing with increasing stage from 90% (95%CI = 73–100) for stage 0 to 0% (95%CI = 0–0) for stage IV. BC relative survival was comparable to sporadic BC in the first two years (2y-RSR = 1.1; 95%CI = 1.1–1.1) and increasing thereafter (5y-RSR = 1.7; 95%CI = 1.6–1.7). For TC (*N* = 56) and EC (*N* = 35), 10y-OS was 87% (95%CI = 74–100) and 64% (95%CI = 38–100), respectively. Overall and cancer-specific mortality in female PHTS patients exceeded general population rates (SMR = 3.7; 95%CI = 2.6–5.0 and SMR = 2.7; 95%CI = 1.6–4.4).

**Conclusions:**

The prognosis of PHTS-related cancers was comparable to the general population. The higher overall mortality in PHTS patients is presumably related to their higher cancer incidence. These findings, and the high survival observed in early-stage cancer, emphasise the importance of recognising PHTS early to facilitate cancer surveillance.

## Background

PTEN hamartoma tumour syndrome (PHTS) is caused by a pathogenic germline variant in the *PTEN* gene. Individuals with PHTS have a high hereditary risk of developing cancer, especially female breast cancer (BC). This BC risk ranges from 54% to 76%, a risk comparable to that conferred by *BRCA1/2* pathogenic variants [1–4]. PHTS patients also have a high risk of endometrial cancer (EC; 6% to 22%) and thyroid cancer (TC; 9% to 21%), and a moderately increased risk of colorectal cancer (CRC), renal cancer (RC) and melanoma (< 10%) [5].

PHTS patients with cancer currently receive standard treatment. However, a range of research suggests that the prognosis and treatment response of PHTS-related cancers might differ from that of sporadic cancers (i.e. without genetic predisposition). For example, somatic *PTEN* loss has been associated with tumour proliferation in EC and triple-negative BC, tumour recurrence and shortened survival in BC patients, and poor RC-specific survival [6–10]. Aberrant somatic PTEN signalling has been associated with BC and RC metastasis and decreased survival [11, 12]. Activation of the somatic PI3K pathway (which includes PTEN) has been shown to contribute to radiotherapy resistance in various cancer types [13, 14], and *PTEN* activity in stromal breast tissue has been associated with diminished radiotherapy and chemotherapy responses [15, 16]. Furthermore, patients with metastatic BC and somatic *PTEN* loss have a shortened survival after trastuzumab treatment [17].

The prognosis and outcomes of treatment in PHTS patients with cancer are unknown, leading to uncertainty whether standard cancer treatment is an appropriate choice for these patients. Therefore, we explored the prognosis of PHTS-related cancers in PHTS patients and the effects of different cancer characteristics and treatments.

## Methods

### Patient selection

Adult PHTS patients were retrospectively recruited via genetic centres, PHTS expert centres, and self-recruitment in Europe during 2019–2023 (Supplementary methods). Patients with a pathogenic or likely pathogenic *PTEN* germline variant, self-reported or reported by the genetic centre (*n* = 512), were included. One additional untested patient who met the genetic testing criteria from the National Comprehensive Cancer Network and had PHTS in first-degree relatives was included [18].

**Fig. 1:**
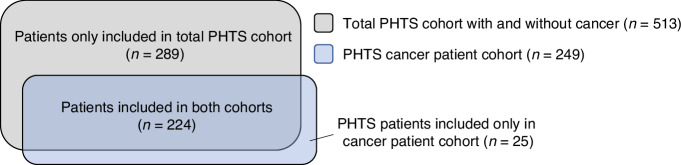
Schematic presentation of the total PHTS cohort and cancer patient cohort. The total PHTS cohort consisted of patients who were recruited based on their PHTS diagnosis, with or without (a history of) cancer (*n* = 513). The PHTS cancer patient cohort consisted of PHTS patients with a history of cancer (*n* = 249). Data for patients only included in the cancer patient cohort were obtained from centres that only provided data on PHTS patients with cancer (*n* = 25).

In total, 513 adult PHTS patients were included in mortality analyses, and 249 PHTS patients with cancer were included in survival analyses (Fig. 1).

This cohort study was approved by the institutional ethics committees, and written informed consent was obtained when indicated by the ethical committee.

### Patient data

Data on cancer characteristics, treatment, follow-up, vital status, PHTS diagnosis, and index status (i.e. an index patient is the first PHTS patient identified in a family; Supplementary methods) were collected from medical files using standardized data dictionaries and patient questionnaires. Data on cause of death were collected from medical files, and death due to cancer was regarded as cancer-specific. For Dutch patients, additional information on vital status, cancers, and treatments was collected via the Dutch Nationwide Pathology Databank (PALGA) and Cancer Registry.

### Statistical analysis

Standardized mortality rates were calculated to compare observed mortality in the total PHTS population with expected mortality using sex, calendar-year, age, and country-standardized population mortality rates (SMR). Observed cancer-specific mortality was compared with expected mortality using cancer-specific population mortality data (SMR_ca_). SMR were stratified because of the different cancer spectra and cancer risks in male and female PHTS patients. As patients develop cancer early in life, the mortality rates were also presented by age group to explore the shape of the cancer risk curves [19].

Survival for the first primary cancer of that specific type was assessed using Kaplan-Meier analyses for PHTS-related cancers (BC, EC, TC, CRC, RC, and melanoma). Overall survival (OS) was calculated from cancer diagnosis to death from any cause or last follow-up, whichever occurred first. For the OS of a specific cancer type, no censoring was applied for subsequent cancer diagnoses. Cancer-specific survival (CSS) was calculated from cancer diagnosis to cancer-specific death or last follow-up, whichever occurred first. Censoring was applied for deaths from other causes. Metastasis-free survival (MFS) was assessed for patients without metastasis at primary cancer diagnosis and was calculated from cancer diagnosis to metastasis, recurrence, death, or last follow-up, whichever occurred first. Disease-free survival (DFS) was assessed for cancer-free patients at the end of treatment and was calculated from end of treatment until diagnosis of metastasis, recurrence, or last known disease-free moment, whichever occurred first. Survival probabilities were only assessed when numbers were sufficient and/or events occurred. To address survival bias, left-truncation was applied using delayed entry times for patients with cancer pre-dating PHTS diagnosis. This decreases the follow-up time after cancer diagnosis for a subset of the population of interest.

Relative risks associated with cancer stage, age, and treatment on survival were analysed using multivariable Cox regression. The proportionality assumption was verified by assessing log-minus-log plots and Schoenfeld residuals.

Relative survival rates were assessed to compare observed survival in PHTS cancer patients with expected survival in the general population using sex, calendar-year, and age-specific reference data (RSR) [19]. Using cancer-specific reference data, the observed survival was compared with expected survival of sporadic cancer (RSR_ca_). Dutch and Belgian cancer-specific reference data were available (46% of cohort), and Dutch reference data were used for other countries [20, 21]. Sensitivity analyses indicated that the Dutch sub-cohort resembled the total cohort (data not shown).

Analyses were performed using RStudio (V.4.1.1). Statistical significance was set at p < 0.05. For SMR, relative risks and RSR, a rate of 1.0 indicates no difference between the reference population or reference category and PHTS population. A rate above (or below) 1.0 indicates a difference between the reference population or reference category and PHTS population. The higher (or lower) the rate, the greater the difference. Statistical significance is reached when the 95% confidence interval (95%CI) does not include 1.0.

## Results

### Overall mortality

Of the 513 patients, including 65% female and 55% index patients, 61 died at median age of 55 years (interquartile range (IQR) 45–63). Of these deaths, 27 were reported as cancer-specific. Female patients had a significantly higher overall mortality than the general population (SMR = 3.7, 95%CI = 2.6–5.0), with the greatest significant increase observed between 50 and 59 years of age (SMR = 5.2, 95%CI = 2.9–8.5) (Table 1). Cancer-specific mortality was also significantly higher in female PHTS patients compared to the general population (SMR_ca_ = 2.7, 95%CI = 1.6–4.4).

**Table 1:** Overall and cancer-specific standardized mortality rates^a^.

	Standardized mortality rate (95%CI), n events				
Age category (y)	Overall		Any cancer		BC
	Female	Male	Female	Male	Female
20–29	2.1 (0.2–7.5), 2	2.4 (0.5–7.1), 3	n/a, 0	12.4 (1.4–44.7), 2	n/a, 0
30–39	4.2 (1.5–9.1), 6	n/a, 0	5.4 (1.4–13.8), 4	n/a, 0	20.5 (5.5–52.6), 4
40–49	2.2 (0.7–5.2), 5	1.5 (0.3–4.5), 3	n/a, 0	1.3 (0.0–7.1), 1	n/a, 0
50–59	5.2 (2.9–8.5), 15	0.7 (0.1–2.7), 2	3.4 (1.4–7.0), 7	0.8 (0.0–4.2), 1	11.9 (4.3–25.9), 6
60–69	3.5 (1.6–6.7), 9	1.6 (0.4–4.1), 4	3.9 (1.4–8.4), 6	0.8 (0.0–4.6), 1	7.4 (0.8–26.8), 2
20–69	3.7 (2.6–5.0), 37	1.2 (0.6–2.2), 12	2.7 (1.6–4.4), 17	1.3 (0.4–3.1), 5	8.1 (4.2–14.2), 12

^a^*n/a* not applicable, *BC* breast cancer.

Standardized mortality rates (SMRs) are presented with corresponding 95% confidence intervals (95%CI) by age groups and sex for the overall PHTS cohort, PHTS cohort with any cancer, and PHTS cohort with breast cancer (BC) (compared to the general population). The number of events is presented for each age category.

### Cancer prognosis, treatment, and mortality

In total, 482 primary cancers were diagnosed in 249 patients (82% female, 66% index). Most patients (89%) had PHTS-related cancers, of which 24% had two different types of cancer and 5% had three types. Females often had BC (147/204) and males often had TC (15/45). PHTS was diagnosed at a median age of 45 years (IQR 34–54) in females and at 50 years (IQR 41–56) in males. Median age at last follow-up was 50 years (IQR 41–59) in females and 58 years (IQR 50–64) in males.

#### Breast cancer

A total of 147 females were included in BC analyses (73% index; Table 2). In 31%, the first primary BC was diagnosed after the PHTS diagnosis (i.e. incident cancer diagnosis). BC was diagnosed at a median age of 41 years (IQR 35–49). The BC stage at diagnosis was mostly stage 0 (33%), stage I (27%), and stage II (23%). Histology was largely carcinoma of no special type (56%) and carcinoma in situ (29%) (Supplementary Table 1). Twenty-five patients died (52% BC-specific) at a median age of 55 years (IQR 50–60).

**Table 2:** Cancer characteristics and treatment characteristics of cancer patient cohorts^a^.

	BC	EC	TC	CRC	RC	Melanoma
Cancer characteristics	Female	Female	Total	Total	Total	Total
Population, No.	147	35	56	22	12	19
Sex, No. (%) female	147 (100)	35 (100)	41 (73)	11 (50)	8 (67)	12 (63)
Sex, No. (%) male	–	–	15 (27)	11 (50)	4 (33)	7 (37)
Index, No. (%)	108 (73)	26 (74)	37 (66)	14 (64)	7 (58)	11 (58)
Incident cancer diagnosis, No. (%)^b^	45 (31)	9 (26)	20 (36)	6 (27)	7 (58)	5 (26)
Ages, median (IQR)						
Cancer diagnosis	41 (35–49)	46 (36–55)	37 (26–46)	60 (53–64)	53 (51–58)	35 (30–42)
PHTS diagnosis	45 (35–53)	51 (41–62)	40 (32–55)	60 (50–65)	51 (45–54)	45 (36–52)
Last follow-up	51 (42–59)	57 (50–66)	51 (41–61)	65 (58–72)	60 (52–64)	51 (45–62)
Follow-up after cancer, median (IQR)	7.2 (3.2–12.9)	6.0 (3.0–16.3)	11.3 (4.5–17.7)	4.2 (2.0–10.0)	2.6 (1.7–7.3)	16.5 (6.2–17.3)
Follow-up after cancer left-truncated analysis, median (IQR)	4.0 (1.6–7.0)	2.3 (1.2–4.8)	5.2 (1.8–9.7)	2.6 (1.0–6.0)	1.9 (0.9–3.6)	5.5 (2.8–10.2)
Death, No. (%)	25 (17)	5 (14)	5 (9)	5 (23)	1 (7)	2 (11)
Cancer-specific, No. (%)	13 (52)	2 (40)	3 (60)	2 (40)	0 (0)	0 (0)
Death age, median (IQR)	55 (50–61)	62 (55–67)	62 (57–64)	73 (59–75)	72	55, 70
Stage at diagnosis, No. (%)						
0	43 (33)	–	–	–	–	–
I	35 (27)	22 (79)	45 (87)	3 (16)	8 (80)	10 (91)
II	30 (23)	4 (14)	3 (6)	5 (26)	1 (10)	1 (9)
III	17 (13)	0 (0)	0 (0)	7 (37)	0 (0)	0 (0)
IV	5 (4)	2 (7)	4 (8)	4 (21)	1 (10)	0 (0)
Unknown^c^	17	7	4	3	2	8
Grade, No. (%)^d^	115 (78)	27 (77)	–	15 (68)	5 (42)	2 (11)
Grade 1	27 (24)	20 (74)	–	0 (0)	2 (40)	2 (100)
Grade 2	53 (46)	6 (22)	–	14 (93)	3 (60)	0 (0)
Grade 3	35 (30)	1 (4)	–	1 (7)	0 (0)	0 (0)
Hormone receptor status, No. (%)^d^	100 (68)	–	–	–	–	–
Hormone receptor ER/PR+	85 (85)	–	–	–	–	–
HER2 status, No. (%)^d^	84 (57)	–	–	–	–	–
HER2+	5 (6)	–	–	–	–	–
Triple-negative (ER/PR/HER2-), No. (%)	12 (14)	–	–	–	–	–
Node-status+ (N1/2/3), No. (%)	42 (29)	1 (3)	10 (18)	8 (36)	1 (8)	0 (0)
Metastasis-status+ (M1), No. (%)	5 (3)	2 (6)	3 (5)	4 (18)	0 (0)	0 (0)

Treatment characteristics, No. (%)^e^						
Surgery^d^	141 (97)	34 (97)	56 (100)	20 (91)	12 (100)	18 (95)
No	3 (2)	2 (9)	2 (4)	2 (10)	2 (17)	0 (0)
Yes	138 (98)	32 (91)	54 (96)	18 (90)	10 (83)	18 (100)
Mastectomy	95 (65)	–	–	–	–	–
Mastectomy bilateral	66 (69)	–	–	–	–	–
Mastectomy unilateral	29 (31)	–	–	–	–	–
Lumpectomy	44 (31)	–	–	–	–	–
Hysterectomy	–	29 (90)	–	–	–	–
Total thyroidectomy	–	–	42 (78)	–	–	–
Hemithyroidectomy	–	–	10 (19)	–	–	–
Total colectomy	–	–	–	2 (11)	–	–
Hemicolectomy	–	–	–	9 (50)	–	–
Nephrectomy unilateral	–	–	–	–	6 (60)	–
Partial nephrectomy	–	–	–	–	4 (40)	–
Skin resection	–	–	–	–	–	18 (100)
Chemotherapy^d^	137 (93)	30 (86)	54 (96)	20 (91)	11 (92)	17 (89)
No	75 (55)	29 (97)	53 (98)	12 (60)	10 (91)	17 (100)
Yes	62 (45)	1 (3)	1 (2)	8 (40)	1 (9)	0 (0)
Neo-adjuvant	24 (39)	0 (0)	0 (0)	2 (25)	0 (0)	0 (0)
Adjuvant	28 (45)	1 (100)	1 (100)	6 (75)	1 (100)	0 (0)
Unknown	10	0	0	0	0	0
Agents used						
Alkaloids	62 (100)	1 (100)	0 (0)	3 (38)	1 (100)	–
Antitumour antibiotics	45 (73)	0 (0)	0 (0)	0 (0)	0 (0)	–
Antimetabolites	25 (40)	0 (0)	0 (0)	8 (100)	1 (100)	–
Platinum-containing	3 (5)	1 (100)	1 (100)	5 (63)	0 (0)	–
Topoisomerase inhibitors	0 (0)	0 (0)	1 (100)	0 (0)	0 (0)	–
Other	1 (2)	0 (0)	0 (0)	0 (0)	0 (0)	–
Radiotherapy^d^	137 (93)	31 (89)	53 (95)	20 (91)	11 (92)	17 (89)
No	73 (53)	22 (71)	52 (96)	20 (100)	11 (100)	17 (100)
Yes	64 (47)	9 (29)	2 (4)	0 (0)	0 (0)	0 (0)
Radioiodine therapy^d^	–	–	34 (61)	–	–	–
No	–	–	2 (6)	–	–	–
Yes	–	–	32 (94)	–	–	–
Hormone therapy^d^	134 (91)	31 (89)	53 (95)	20 (91)	11 (92)	18 (95)
No	69 (51)	30 (97)	53 (100)	20 (100)	11 (100)	18 (100)
Yes	65 (49)	1 (3)	0 (0)	0 (0)	0 (0)	0 (0)
Agents used						
Progestogen	0 (0)	1 (100)	–	–	–	–
Antioestrogen	40 (62)	0 (0)	–	–	–	–
Aromatase-inhibitor	16 (25)	0 (0)	–	–	–	–
LHRH-agonist	2 (3)	0 (0)	–	–	–	–
Other	7 (11)	0 (0)	–	–	–	–
Immune therapy^d^	130 (88)	31 (89)	54 (96)	20 (91)	11 (92)	18 (95)
No	122 (94)	31 (100)	53 (98)	20 (100)	11 (100)	18 (100)
Yes	8 (6)	0 (0)	1 (2)	0 (0)	0 (0)	0 (0)
Agents used						
Trastuzumab	6 (75)	–	–	–	–	–
Atezolizumab	2 (25)	–	–	–	–	–
Lenvatinib	0 (0)	–	1 (100)	–	–	–

^a^*BC* breast cancer, *EC* endometrial cancer, *TC* thyroid cancer, *CRC* colorectal cancer, *RC* renal cancer, *IQR* interquartile range.

^b^Cancer diagnosed after DNA diagnosis of PHTS is an incident cancer diagnosis. BC was diagnosed a median of 0.9 year (IQR 0.0–6.4) before PHTS. EC was diagnosed 1.4 years (IQR 0.0–9.9) before PHTS. TC was diagnosed 1.2 years (IQR 0.0–12.0) before PHTS. CRC was diagnosed 0.0 years (IQR 0.0–1.4) before PHTS.

^c^‘Unknown’ stage is not included in the percentages.

^d^Availability of information.

^e^The frequencies for treatment are not mutually exclusive as participants could have multiple treatments and multiple agents used per treatment type.

All patients, except three with stage IV BC, underwent surgical treatment (96%; Table 2) including bilateral mastectomy in 47% of patients (35% of patients with an incident BC diagnosis), unilateral mastectomy in 21% (7% in incident BC), and lumpectomy in 31% (24% in incident BC). Overall, 58% received both systemic and non-systemic treatments, and 31% only non-systemic. Treatment initiation was related to tumour characteristics, e.g. 0%–39% received chemotherapy for stages 0–I versus 77%–80% for stages II–IV.

**Fig. 2:**
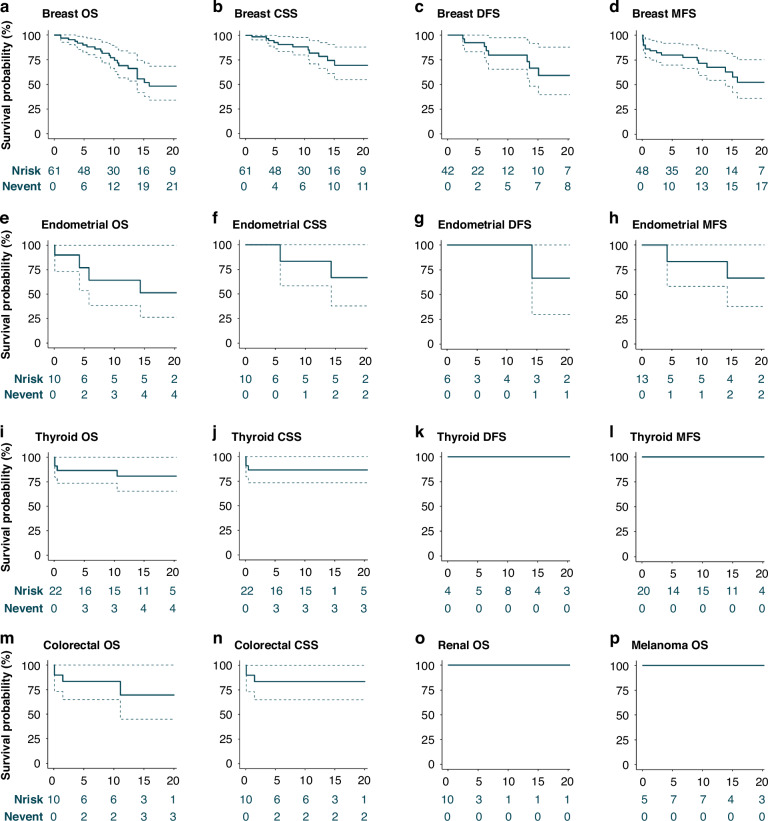
Survival per cancer type. The survival probability per cancer type in percentages (%) on the y-axis is presented for the left-truncated analyses. The x-axis represents the number of years after cancer diagnosis. The dashed lines represent 95% confidence intervals. The number of patients at risk (Nrisk) and the cumulative number of events (Nevent) are presented in the risk tables. Survival for breast and endometrial cancer is presented only for females. Overall survival (OS) is presented for female breast, endometrial, thyroid, colorectal, renal cancer, and melanoma (**a**, **e**, **i**, **m**, **o**, **p**). The cancer-specific survival (CSS) is presented for female breast, endometrial, thyroid, and colorectal cancer (**b**, **f**, **j**, **n**). Disease-free survival (DFS) is presented for female breast, endometrial, and thyroid cancer (**c**, **g**, **k**). The metastasis-free survival (MFS) is presented for breast, endometrial, and thyroid cancer (**d**, **h**, **l**). The 5- and 10-year survival per cancer type is presented in Supplementary Table 2.

The 5y- and 10y-OS were 90.0% (95%CI = 82.7–98.0) and 77.1% (95%CI = 66.3–89.6), respectively. The median OS was 21 years. For invasive BC, the 5y- and 10y-OS were 83.6% (95%CI = 73.7–94.8) and 67.3% (95%CI = 54.3–83.4), respectively. For stages 0–IV the 5y-OS was 100.0% (95%CI = 100.0–100.0), 93.3% (95%CI = 81.5–100.0), 88.9% (95%CI = 70.6–100.0), 80.0% (95%CI = 51.6–100.0), and 33.3% (95%CI = 6.7–100.0), respectively. The 10y-OS by stage was 90.0% (95%CI = 73.2–100.0), 83.0% (95%CI = 63.5–100.0), 74.1% (95%CI = 48.4–100.0), 51.4% (95%CI = 26.4–100.0) and 0.0% (95%CI = 0.0–0.0), respectively. CSS and DFS were higher, whereas MFS was lower than OS (Fig. 2; Supplementary Table 2).

BC patients who underwent mastectomy appeared to have better survival compared to those who underwent breast-conserving surgery (HR_unilateral_ = 0.8, 95%CI = 0.2–3.7 and HR_bilateral_ = 0.2, 95%CI = 0.03–0.9; Supplementary Fig. 1). Poorer survival was observed in patients treated with chemotherapy or radiotherapy compared to those treated without chemotherapy or radiotherapy (HR = 5.4, 95%CI = 1.1–25.3 and HR = 3.1, 95%CI = 0.8–11.9), respectively. All patients with hormone receptor-positive BC who did not receive hormone therapy survived ten years after diagnosis. Overall, patients who received systemic therapy had lower survival rates compared to those receiving non-systemic treatment (HR = 7.6, 95%CI = 1.0–59.7). Results of multifactorial analyses of hormone therapy, mastectomy, and systemic treatment, including nodal-status or age at diagnosis, were similar (data not shown). Multifactorial analyses of chemotherapy were not possible.

Compared to the general population, BC-specific mortality was significantly higher in PHTS patients (SMR_ca_ = 8.1, 95%CI = 4.2–14.2) and a trend towards stronger increase at younger age was observed (Table 1).

**Fig. 3:**
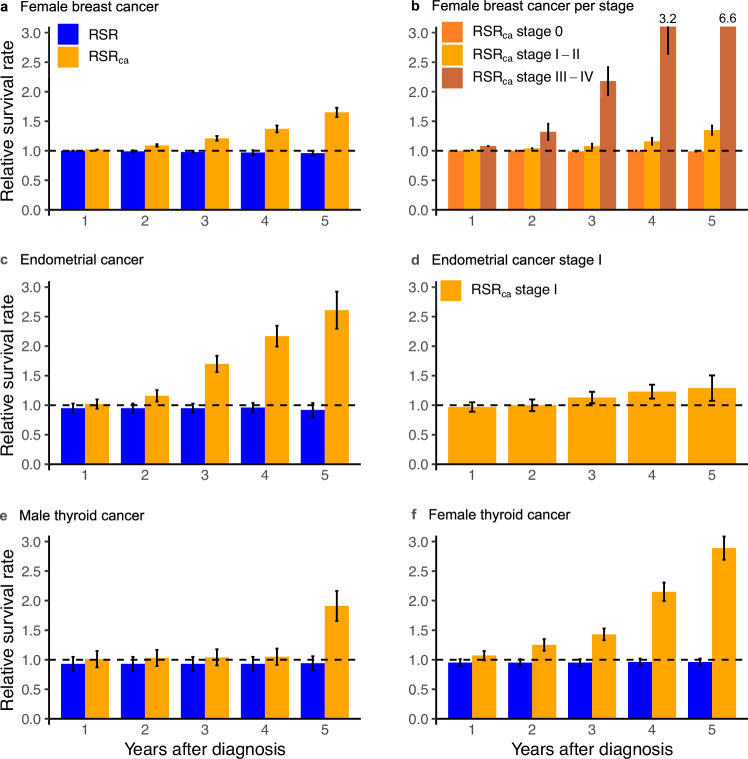
Relative survival rates for breast cancer, endometrial cancer, and thyroid cancer. Comparison of observed survival in PHTS cancer patients with expected survival in the general population is presented as the relative survival rates (RSR). Comparison of observed survival in PHTS cancer patients with expected survival in sporadic cancer is presented as RSR_ca_. RSR is presented on the y-axis for 1 to 5 years after cancer diagnosis (x-axis). Error bars represent the 95% confidence intervals. For breast and endometrial cancer, the RSR_ca_ is additionally presented by stage (**b, d**). Breast cancer stage 0 refers to carcinoma in situ (**b**).

For patients with PHTS, relative survival after a BC diagnosis was comparable to survival in the general population (Fig. 3a). Until two years post-diagnosis, relative survival was also comparable to sporadic BC (1y-RSR_ca_ = 1.0, 95%CI = 1.0–1.0 and 2y-RSR_ca_ = 1.1, 95%CI = 1.1–1.1). Thereafter, it gradually increased to 1.7 (95%CI = 1.6–1.7) at five years post-diagnosis (Fig. 3a). For stage 0 (carcinoma in situ), relative survival was comparable up to five years post-diagnosis (RSR_ca_ = 1.0, 95%CI = 1.0–1.0). Comparable relative survival was observed for stage I-II BC until three years post-diagnosis and for stage III-IV BC until one year post-diagnosis, and increased thereafter (Fig. 3b).

#### Endometrial cancer

Thirty-five females with EC were included (74% index; Table 2), of whom 20% previously used tamoxifen. Of EC diagnoses, 26% were incident. EC was diagnosed at a median age of 46 years (IQR 36–55), PHTS at 51 years (IQR 41–62), and last follow-up was at 57 years (IQR 50–66). EC was mostly stage I (79% of patients) and of endometrioid histology (69%; Supplementary Table 1). Most patients underwent surgical treatment, including hysterectomy in 90%. Five patients died at a median age of 62 years (IQR 55–67), of which two EC-specific deaths occurred between 60 and 69 years.

The 5y- and 10y-OS were 77.1% (95%CI = 53.5–100.0) and 64.3% (95%CI = 38.5–100.0), respectively. The 5y- and 10y-OS were 80.0% (95%CI = 51.6–100.0) for patients with stage I EC. Numbers were too small for specific stage II-IV analyses. Two EC-specific deaths were observed, reflected in the high 5y- and 10y-CSS (Fig. 2f; Supplementary Table 2). None of the patients without EC after treatment (*n* = 6) had metastasis or recurrence after ten years of follow-up (DFS = 100%, 95%CI = 100–100). The 5y- and 10y-MFS were both 83.3% (95%CI = 58.3–100.0).

Relative survival after EC was comparable to the general population (Fig. 3c). Relative survival after EC was also comparable to sporadic EC until one year post-diagnosis (1y-RSR_ca_ = 1.0, 95%CI = 0.9–1.1) and gradually increased to 2.6 (95%CI = 2.3–2.9) five years post-diagnosis (Fig. 3c). Stage I-specific relative survival was comparable to sporadic EC (1y-RSR_ca_ = 1.0, 95%CI = 0.9–1.1 to 5y-RSR_ca_ = 1.3, 95%CI = 1.1–1.5) (Fig. 3d).

#### Thyroid cancer

Fifty-six patients with TC were included (66% index, 73% female; Table 2). Of TC diagnoses, 36% were incident. TC was diagnosed at a median age of 37 years (IQR 26–46). TC was mostly stage I (87%) and follicular (36%) or papillary histology (41%) (Supplementary Table 1). Most patients underwent surgical treatment including total (78%) or hemithyroidectomy (19%) and received additional radioiodine therapy (94%). Five patients died (3 TC-specific) at a median age of 62 years (IQR 57–64). All three TC-deaths occurred within one year of an initial stage IV TC diagnosis.

The 5y- and 10y-OS for TC were both 86.6% (95%CI = 73.5–100.0) and were comparable by sex (data not shown). Stage I-specific OS was 100% after ten years. No recurrences or metastases were observed after the TC diagnosis and treatment (Fig. 2j–l; Supplementary Table 2).

Relative survival after TC was comparable to the general population for males and females (Fig. 3e, f). Relative survival after TC was also comparable to sporadic TC until three years post-diagnosis for males (1-3y-RSR_ca_ = 1.0, 95%CI = 0.9–1.2) and until one year post-diagnosis for females (1y-RSR_ca_ = 1.1, 95%CI = 1.0–1.2). Thereafter, the RSR_ca_ gradually increased to 1.9 (95%CI = 1.7–2.2) for males and to 2.9 (95%CI = 2.7–3.1) for females five years post-diagnosis. Stage-specific analyses were not possible due to small numbers.

#### Colorectal cancer

Twenty-two patients with CRC were included (64% index, 50% female; Table 2). Of CRC diagnoses, 27% were incident. CRC was diagnosed at a median age of 60 years (IQR 53–64). Stage at diagnosis was mostly stage III (37%). Most patients underwent surgical treatment, consisting of hemicolectomy in 50% of patients. Eight patients received additional chemotherapy (75% adjuvant), mostly for stage III CRC (75%). Five patients died (2 CRC-specific) at a median age of 73 years (IQR 59–75). Both CRC-specific deaths occurred within five years of diagnosis. The 5y- and 10y-OS were both 83.6% (95%CI = 64.9–100.0) (Fig. 2n; Supplementary Table 2).

#### Renal cancer

Twelve patients with RC were included (58% index, 63% female; Table 2). Of RC diagnoses, 58% were incident. RC was diagnosed at a median age of 53 years (IQR 51–58). Most patients had stage I RC (80%). Most patients underwent surgical treatment (83%), including unilateral (60%) and partial nephrectomy (40%). One patient died, which was not RC-specific.

#### Melanoma

Nineteen patients with melanoma were included (58% index, 63% female; Table 2). Of melanoma diagnoses, 26% were incident. Melanoma was diagnosed at a median age of 35 years (IQR 30–42). Most patients had stage I melanoma (91%) and all (100%) received surgical treatment only. Two patients died from other causes.

## Discussion

Rare hereditary syndromes associated with predisposition to cancer can offer valuable insights into sporadic cancers which often have somatic (i.e. non-germline) variants in the gene associated with hereditary predisposition. Somatic variants of the *PTEN* tumour suppressor gene are common in sporadic cancer, and the effect of these variants on the prognosis of sporadic cancers has been debated for years [22, 23]. Unlike well described hereditary cancer risk syndromes such as *BRCA1/2* [24–26], this is the first study to evaluate the prognosis of cancer in patients with germline pathogenic variants in *PTEN* (i.e. in individuals with PHTS). Unlike findings from previous studies on somatic *PTEN* variants in sporadic cancer [6–17], this study found no indication of germline pathogenic variants in *PTEN* adversely affecting cancer prognosis.

### Breast cancer

In our study, the 5y- and 10y-OS of PHTS patients with BC was 90.0% and 77.1%, respectively. This is comparable to outcomes for sporadic BC (75%–92% and 66%–80%) [21, 27]. Consistent with sporadic BC, survival decreased with increasing stage in PHTS patients with BC. The 5y-OS was 93.3%, 88.9%, 80.0%, 33.3%; and the 10y-OS was 83.0%, 74.1%, 51.4%, 0.0% for stage I-IV BC in PHTS patients, compared to 5y-OS: 99%, 92%, 77%, 31%; and 10y-OS: 96%, 85%, 64%, 12% for BC in the general population [21].

Relative survival for PHTS patients with BC was comparable to patients with sporadic BC for the first two years after diagnosis. Thereafter, it gradually increased to 1.7 (95%CI = 1.6–1.7) at five years post-diagnosis, indicating relatively favourable outcomes compared to sporadic BC. Comparable relative survival initially followed by slightly increased relative survival compared to sporadic BC was observed for both stage I-II and stage III-IV BC in PHTS patients. In these analyses, relative survival may have been overestimated due to survival bias. Furthermore, more aggressive treatment, cancer prevention strategies, and healthy screener effects might have influenced these results, which requires cautious interpretation of these results [28–30].

Of note, (bilateral and unilateral) mastectomy rates were higher in PHTS patients with BC than in patients with sporadic BC (65% vs. 15–30%) and were comparable to rates in patients with a *BRCA1/2* pathogenic variant (59%) [31–33]. Amongst PHTS patients with BC, better survival was observed with unilateral and bilateral mastectomy than with breast-conserving surgery (HR_unilateral_ = 0.8, 95%CI = 0.2–3.7 and HR_bilateral_ = 0.2, 95%CI = 0.03–0.9), consistent with some *BRCA1/2* reports while other reports demonstrated no difference in survival [34, 35]. However, survival after unilateral mastectomy in PHTS patients with BC was not statistically significantly increased, and the intent of surgery was not always known. Furthermore, this effect could be related to the high risk of second primary BC in PHTS patients [36]. Thus, further confirmation using a larger cohort is desirable. While awaiting such results, our findings support counselling of female PHTS patients on the possibility of bilateral mastectomy as cancer treatment for the affected breast and as risk-reducing surgery for the contralateral breast.

Our results suggest lower survival in PHTS patients with BC who received chemotherapy or radiotherapy compared to patients who did not (HR = 5.4, 95%CI = 1.1–25.3 and HR = 3.1, 95%CI = 0.8–11.9, respectively). However, this is most likely related to underlying prognostic factors which indicated a need for these treatment modalities [37]. Therefore, additional data are needed and no new recommendations can be made on use of chemotherapy or radiotherapy for BC treatment in PHTS patients.

Although survival (i.e. percentage of people alive at selected time-points after diagnosis) in PHTS patients with BC was no worse than in sporadic BC patients, BC mortality (i.e. number of deaths from BC in the PHTS population) and overall mortality (i.e. number of deaths from any cause in the PHTS population) in female PHTS patients was increased compared to the general population (SMR_ca_ = 3.7, 95%CI = 2.6–5.0), and was to an extent comparable to that in female *BRCA1/2* patients (SMR_ca_ = 3.2, 96%CI 2.0–5.3) [24]. This presumably relates to the higher BC incidence in PHTS patients and might be overestimated due to ascertainment bias [5]. More PHTS patients develop cancer due to a high risk of cancer compared to the general population. Despite the comparable prognosis compared to sporadic cancer, the high cancer incidence presumably contributes to increased mortality, which likely results in excess mortality in PHTS patients. Numbers for SMR subgroup analyses by age were low and should therefore be interpreted with caution.

### Other PHTS-related cancers

Our findings in other PHTS-related cancers were similar to findings in BC. Most patients with EC, TC, RC, and melanoma had early-stage disease (stage I) whereas half of the patients with CRC had stage III-IV disease at diagnosis. Histology, grade, and hormone status distribution across cancer types is similar to the general population, except for RC which is potentially due to low number of events [21]. Treatments for these PHTS-related cancers were generally comparable to treatments for sporadic cancers. Most PHTS patients with EC underwent hysterectomy (90% vs. 97% of patients with sporadic EC), and a minority underwent radiotherapy (29% vs. 31%) [38]. TC treatment in PHTS patients was comparable to sporadic TC for total thyroidectomy (78% vs. 85%), with a higher frequency of radioiodine therapy in PHTS patients (94% vs. 72%) [39]. CRC treatment in PHTS patients was comparable for chemotherapy (40% vs. 35%), but more PHTS patients than sporadic CRC patients underwent surgery (90% vs. 75%) [40]. The frequency of surgery in PHTS patients with RC was higher although less extensive than in patients with sporadic RC (unilateral nephrectomy 60% vs. 82%; partial nephrectomy 40% vs. 14%), probably reflecting the early stage at diagnosis. The frequency of surgery was comparable for PHTS patients with melanoma and sporadic melanoma (100% vs. 95%) [41, 42].

As seen for PHTS patients with BC, OS was no worse in PHTS-related cancers than in sporadic cancers. The 5y-OS and 10y-OS for EC (77.1% and 64.3%) and TC (both 86.6%) were comparable to sporadic cancer (EC: 5y-OS 79%–82% and 10y-OS 76%; TC: 5y-OS 85% and 10y-OS 80%) [21, 43, 44], with no indication of worse relative survival. The 5y- and 10y-OS for CRC (both 83.6%) were higher than reported for sporadic CRC (5y-OS 67% and 10y-OS 61%), potentially due to a lower proportion of advanced CRC in PHTS patients, who may undergo regular colonoscopies for polyps from an early age [21, 45, 46]. However, stage-specific analyses could not be performed due to low numbers of PHTS patients with CRC. For RC and melanoma, no deaths occurred within ten years, suggesting favourable prognoses. Although death from any cause was taken into account, no censoring was applied for diagnosis of other cancers in the follow-up, which might have led to underestimation of these OS estimates.

### Limitations

The rarity of PHTS makes collecting sufficient patient data challenging. Potential survival bias was addressed by applying left-truncated analyses and excluding patients from institutes where information sharing from deceased patients was impossible. Although we collected a substantial cohort of PHTS cancer patients, including patients from sixteen countries, the number remained limited for less prevalent cancers and multifactor analyses of cancer characteristics. While lymph node status was assessed for BC in our study, other factors (including hormone and HER2 receptor status, physical activity, comorbidities, and other primary cancers) that could influence survival outcomes were not sufficiently available or not collected [47–49]. A matched case-control study could potentially provide more statistical power to assess combinations of prognostic factors in PHTS compared to cancer in the general population [27]. Although a follow-up study using a larger prospective cohort is desirable, short-term realization is not feasible due to the rarity of PHTS.

### Significance

For high hereditary cancer risk syndromes, it is crucial to understand cancer prognosis, treatment, and mortality to optimize patient care. Our study shows that the prognosis of cancer in PHTS patients is no worse than for comparable cancers in the general population (sporadic cancers). Thus, unlike previous studies on somatic *PTEN* variants in sporadic cancer [6–17], our findings suggest that pathogenic germline *PTEN* variants do not worsen cancer prognosis. Our results also support the view that standard cancer treatment is appropriate for PHTS patients with cancer. These findings may provide some reassurance to PHTS patients. For PHTS patients with BC, bilateral mastectomy may result in a higher survival compared to breast-conserving surgery, but further evaluation is required.

Although the prognosis of PHTS-associated cancers (BC, EC, TC, CRC, RC, and melanoma) was comparable to sporadic cancers, overall and cancer-specific mortality was higher in female PHTS patients than the general population. This is presumably related to the high incidence of cancer in female PHTS patients. These observations and the favourable outcomes of early-stage cancer in PHTS patients highlight the importance of a timely diagnosis of PHTS and initiation of appropriate surveillance and/or risk-reducing surgery in patients with PHTS.

## Supplementary information

Supplementary methods

## Data Availability

The individual patient data that support the findings of this study are not openly available due to privacy and ethical reasons. Aggregate study data are available from the corresponding author upon reasonable request. Data are located in controlled access data storage at Radboud university medical centre.
