# Supplementary material for: Cancer prognosis and treatment results in patients with PTEN Hamartoma Tumour Syndrome (PHTS)—a European cohort study
Source: BJC Rep. 2025 Jun 4;3:42. doi: 10.1038/s44276-025-00157-y (PMC12137667; doi:10.1038/s44276-025-00157-y)
Supplement: Supplementary file 1 — Supplementary methods [file 44276_2025_157_MOESM1_ESM.pdf]

Supplementary methods

Patient recruitment

PHTS patients were recruited via 30 medical centres in 16 European countries or self-recruitment during 2019-2023. Self-recruitment entailed patients registering themselves via a website for our study, which was disseminated with the support of patient societies. After obtaining consent, medical data were requested from the medical centres listed by the patient. Double inclusion was checked by comparing potential duplicate cases with similar data points and by contacting the medical centres. Selection in type of patients per centre (e.g. selection in age, cancer type, vital status) was assessed. Centres were excluded from survival analyses when collecting data from deceased patients was not allowed, and only patients alive at moment of inclusion could be included (n=1 centre; 13 patients).

PHTS index status

The index patient is the first patient in a family to be diagnosed with PHTS and to undergo genetic germline testing of *PTEN* based on clinical suspicion. PHTS patients can present with a broad and variable phenotypic spectrum, including macrocephaly, neurodevelopmental delay, cutaneous lesions, vascular malformations, and benign and malignant tumours. This means that the index status is not solely related to history of cancer. Of the PHTS index patients, 48% were diagnosed with cancer before the PHTS diagnosis. Similarly, non-index patients can also have cancer before the PHTS diagnosis (21% of the non-index patients).

Supplementary data

Supplementary Table 1. Histology and characteristics of first primary PHTS-related cancer.

Cancer histology	No. (%)	Grade, No. (%)				Hormone receptor status, No. (%)		HER2 status, No. (%)		Triple-negative, No. (%)
		No.[a]	1	2	3	No.[a]	ER/PR+	No.[a]	HER2+	ER/PR/HER2-
**Breast cancer (including in situ)**	**147**	**115**	**27**	**53**	**35**	**100**	**85 (85)**	**84**	**5 (6)**	**12 (14)**
Carcinoma NST	83 (56)	66	8 (12)	35 (53)	23 (35)	73	59 (81)	65	5 (8)	8 (12)
In situ	43 (29)	31	14 (45)	12 (39)	5 (16)	11	8 (73)	4	0 (0)	0 (0)
Lobular	5 (3)	5	1 (20)	4 (80)	0 (0)	5	4 (80)	4	0 (0)	0 (0)
Adenocarcinoma	4 (3)	4	0 (0)	0 (0)	4 (100)	4	0 (0)	3	0 (0)	2 (67)
Not specified/other	12 (8)	9	4 (45)	2 (22)	3 (33)	9	5 (56)	8	0 (0)	2 (25)
**Endometrial cancer**	**35**	**27**	**20**	**6**	**1**	**-**	**-**	**-**	**-**	**-**
Endometrioid	24 (69)	19	12 (63)	6 (32)	1 (5)	-	-	-	-	-
Adenocarcinoma	6 (17)	4	4 (100)	0 (0)	0 (0)	-	-	-	-	-
Adenosquamous	1 (3)	1	1 (100)	0 (0)	0 (0)	-	-	-	-	-
Not specified	4 (11)	3	3 (100)	0 (0)	0 (0)	-	-	-	-	-
**Thyroid cancer**	**56**	**-**	**-**	**-**	**-**	**-**	**-**	**-**	**-**	**-**
Follicular	20 (36)	-	-	-	-	-	-	-	-	-
Follicular papillary	8 (14)	-	-	-	-	-	-	-	-	-
Papillary	23 (41)	-	-	-	-	-	-	-	-	-
Anaplastic	3 (5)	-	-	-	-	-	-	-	-	-
Medullary	1 (2)	-	-	-	-	-	-	-	-	-
Not specified	1 (2)	-	-	-	-	-	-	-	-	-
**Colorectal cancer**	**22**	**15**	**0**	**14**	**1**	**-**	**-**	**-**	**-**	**-**
Adenocarcinoma	17 (77)	12	-	11 (92)	1 (8)	-	-	-	-	-
Mucinous adenocarcinoma	3 (14)	2	-	2 (100)	0 (0)	-	-	-	-	-
Not specified	2 (9)	1	-	1 (100)	0 (0)	-	-	-	-	-
**Renal cancer**	**12**	**5**	**2**	**3**	**0**	-	-	-	-	-
Papillary	5 (42)	2	1 (50)	1 (50)	-	-	-	-	-	-
Clear cell	4 (33)	3	1 (33)	2 (67)	-	-	-	-	-	-
Renal cell NOS	2 (17)	0	0 (0)	0 (0)	-	**-**	**-**	**-**	**-**	**-**
Adenocarcinoma	1 (8)	0	0 (0)	0 (0)	-	-	-	-	-	-
**Melanoma**	**19**	**2**	**2**	**0**	**0**	-	-	-	-	-
Melanoma NOS	14 (74)	1	1 (100)	-	-	-	-	-	-	-
Superficial spreading melanoma	3 (16)	0	0 (0)	-	-	-	-	-	-	-
Squamous	1 (5)	1	1 (100)	-	-	-	-	-	-	-
Acral lentiginous melanoma	1 (5)	0	0 (0)	-	-	-	-	-	-	-

NST = no special type; NOS = not otherwise specified

[a] Availability of information

Supplementary Table 2. Survival per cancer type. [a]

Cancer type	Survival type [b]	Time (years)	Number at risk	Cumulative number of events	Survival probability (%)	95%CI
Breast	OS	5	48	6	90.0	82.7-98.0
		10	30	12	77.1	66.3-89.6
	CSS	5	48	4	92.9	86.4-99.9
		10	30	6	88.5	80.1-97.7
	DFS	5	22	2	92.6	83.2-100.0
		10	12	5	79.9	65.5-97.4
	MFS	5	35	10	80.1	69.8-92.0
		10	20	13	71.7	59.4-86.4
Endometrial	OS	5	6	2	77.1	53.5-100.0
		10	5	3	64.3	38.5-100.0
	CSS	5	6	0	100.0	100.0-100.0
		10	5	1	83.3	58.3-100.0
	DFS	5	3	0	100.0	100.0-100.0
		10	4	0	100.0	100.0-100.0
	MFS	5	5	1	83.3	58.3-100.0
		10	5	1	83.3	58.3-100.0
Thyroid	OS	5	16	3	86.6	73.5-100.0
		10	15	3	86.6	73.5-100.0
	CSS	5	16	3	86.6	73.5-100.0
		10	15	3	86.6	73.5-100.0
	DFS	5	5	0	100.0	100.0-100.0
		10	8	0	100.0	100.0-100.0
	MFS	5	14	0	100.0	100.0-100.0
		10	15	0	100.0	100.0-100.0
Colorectal	OS	5	6	2	83.6	64.9-100.0
		10	6	2	83.6	64.9-100.0
	CSS	5	6	2	83.6	64.9-100.0
		10	6	2	83.6	64.9-100.0
Renal	OS	5	3	0	100.0	100.0-100.0
		10	1	0	100.0	100.0-100.0
Melanoma	OS	5	7	0	100.0	100.0-100.0
		10	7	0	100.0	100.0-100.0

[a] The survival probability per cancer type in percentages (%) with 95% confidence intervals (95%CI) is presented for the left-truncated analyses. The time represents the number of years after cancer diagnosis. Survival for breast and endometrial cancer is presented only for females.

[b] Overall survival (OS); cancer-specific survival (CSS); disease-free survival (DFS); metastasis-free survival (MFS)

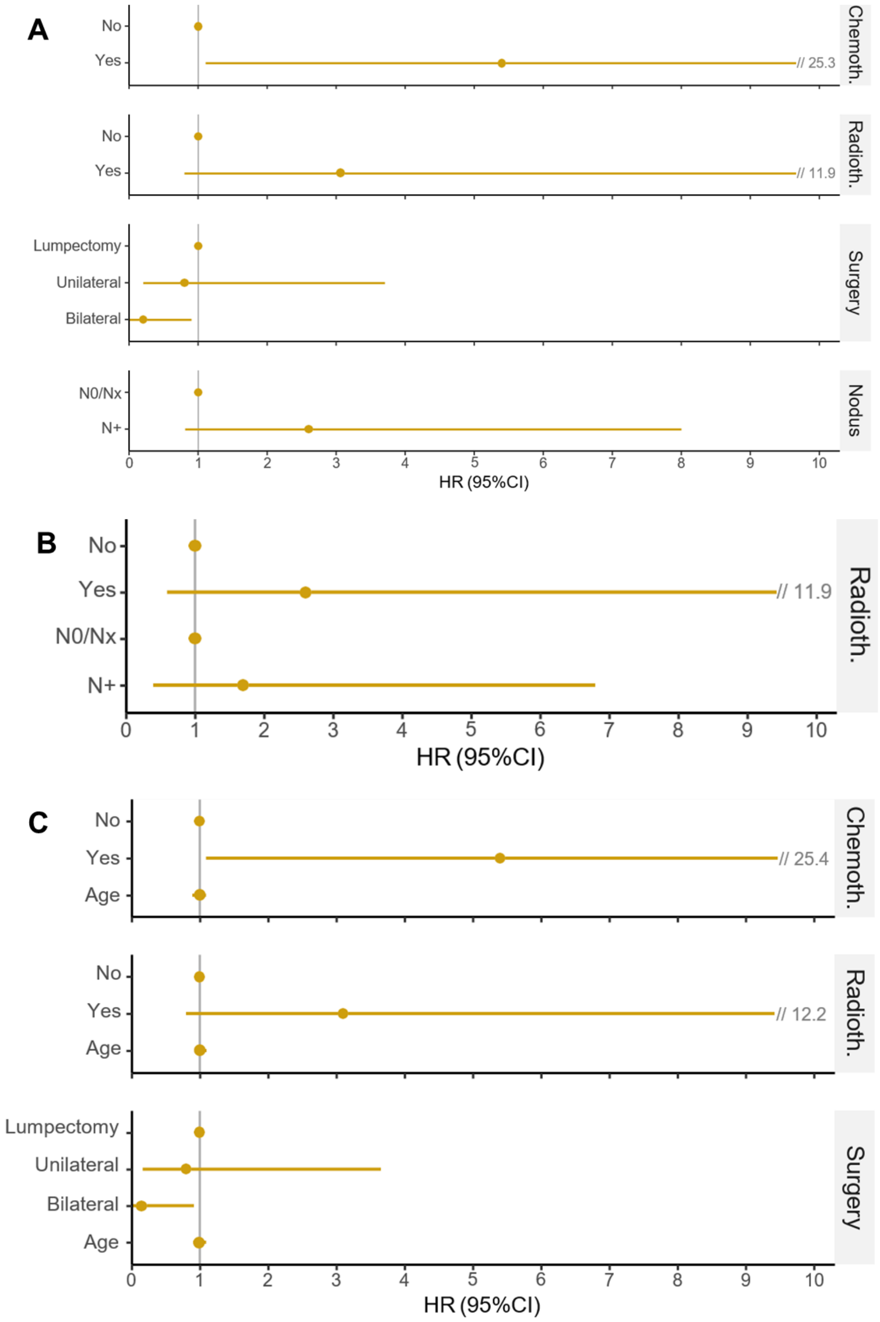

Supplementary Figure 1. Relative risks for survival after breast cancer diagnosis in female PHTS patients.

Hazard ratios (HRs) within 10 years after breast cancer diagnosis are presented with corresponding 95% confidence intervals (95%CI) for the univariate model (**A**); the multivariable Cox regression model including a single treatment factor and node-status (N) (**B**); the multivariable Cox regression model including a single treatment factor and age (**C**). The HR represents the age increment of 1 year (y). Results are presented for the left-truncation corrected BC cohort. For chemotherapy (Chemoth.) and radiotherapy (Radioth.), the reference category is ‘no’. Surgery is analysed as a time-dependent variable where a lumpectomy is the reference category. The unilateral and bilateral mastectomy groups are compared to this reference group. For nodal-status, no nodal metastasis (N0/Nx) is the reference category. Any positive lymph node (N+) is compared to this reference group.
